# Alkaloids Reported in the Genus *Griffinia* (Amaryllidaceae): Phytochemical Evidence and Current Limitations in Anticancer Interpretation

**DOI:** 10.3390/ijms27198466

**Published:** 2026-09-23

**Authors:** Maykon Jhuly Martins de Paiva, Caroline Priscila Furlanetto, Elis Ferreira Garcia, Layra Eugenio Pedreira, Walmirton Bezerra D’Alessandro, Sávia Denise Silva Carlotto Herrera, Renata Ferreira Diogo De Paiva, André Luiz Gomide de Morais, Mateus Silva Santos, Adolpho Dias Chiacchio, Taides Tavares dos Santos, I Made Dwi Mertha Adnyana, Márcio Trevisan

**Affiliations:** 1 Natural Products Laboratory, Department of Pharmacy, Health Sciences School, University of Brasília (UnB), Campus Darcy Ribeiro, Asa Norte, Brasilia 70910-900, Brazil; 2 School of Medicine, University of Gurupi (UnirG), Paraíso do Tocantins Campus, Paraíso do Tocantins 77545-208, Brazil; caroline.furlanetto@unirg.edu.br (C.P.F.); elisf.garcia2702@gmail.com (E.F.G.); layra.e.pedreira@unirg.edu.br (L.E.P.); walmirton@unirg.edu.br (W.B.D.); saviaherrera@unirg.edu.br (S.D.S.C.H.); renataferreiradiogodepaiva@gmail.com (R.F.D.D.P.); mateus.s.santos@unirg.edu.br (M.S.S.); adolphochiacchio@unirg.edu.br (A.D.C.); 3 School of Medicine, University of Gurupi (UnirG), Gurupi 77400-000, Brazil; andreluiz@unirg.edu.br; 4 Graduate Program in Animal Health and Public Health in the Tropics, Faculty of Health Sciences, Federal University of Northern Tocantins (UFNT), Araguaína 77814-350, Brazil; taides.santos@ufnt.edu.br; 5 Department of Microbiology and Parasitology, Faculty of Medicine and Health Sciences, Universitas Jambi, Jambi 36361, Indonesia; i.madedwimertha@unja.ac.id; 6 Graduate Program in Environmental Sciences, Federal University of Tocantins (UFT), Palmas 77001-090, Brazil; marciotrevi@mail.uft.edu.br

**Keywords:** *Griffinia*, Amaryllidaceae alkaloids, phytochemical diversity, anticancer activity, natural products, structure–activity relationship

## Abstract

Natural products continue to provide pharmacologically relevant scaffolds for anticancer drug discovery, with alkaloids from the Amaryllidaceae family among the most structurally diverse and biologically investigated classes of plant secondary metabolites. Within this family, the Brazilian genus *Griffinia*—comprising approximately 21 species predominantly endemic to the Atlantic Forest and Cerrado—remains substantially underrepresented in the phytochemical and pharmacological literature, with fewer than five species subjected to any level of biological evaluation and no genus-wide mechanistic profile available to date. This critical review specifically examines what the current literature reports regarding the anticancer relevance of *Griffinia* alkaloids, addressing a gap that has not been systematically investigated in prior Amaryllidaceae reviews. Six structural classes—lycorine-, galanthamine-, crinine-, hemanthamine-, montanine-, and homolycorine-type alkaloids—have been detected in *Griffinia* species primarily through gas chromatography–mass spectrometry and liquid chromatography–mass spectrometry profiling; however, isolation and direct biological validation have been reported for only one species, *Griffinia gardneriana*, in which lycorine and related compounds exhibit cytotoxic and genotoxic activities associated with caspase-3-mediated apoptosis. This finding constitutes the sole genus-specific mechanistic anchor currently available. The broader biological literature on Amaryllidaceae alkaloids—encompassing apoptosis induction, cell cycle arrest, redox imbalance, and modulation of phosphoinositide 3-kinase/protein kinase B (PI3K/Akt), nuclear factor kappa B (NF-κB), and mitogen-activated protein kinase (MAPK) signaling—is explicitly used here as contextual evidence for the structural classes detected in *Griffinia*, not as proof of genus-level activity. This distinction is central to the evidentiary framework of the review and is maintained throughout. Approximately 70% of evaluated Amaryllidaceae species lack comprehensive phytochemical and biological characterization, a limitation that is directly reflected in *Griffinia*. Future research priorities include rigorous compound isolation and structural elucidation across underexplored species, genus-specific bioactivity testing in well-defined cancer models, and mechanism-oriented studies anchored in direct experimental evidence rather than structural analogies. Semi-synthetic optimization and multi-omic approaches represent longer-term but tractable avenues once the primary pharmacological characterization is established.

## 1. Introduction

Cancer is one of the leading health challenges of the 21st century. Global estimates indicate that the worldwide burden will reach 35 million new cases by 2050 [1]. Despite advances in diagnosis, surgery, radiotherapy, and systemic treatment, disease recurrence, metastatic spread, and therapeutic resistance continue to limit disease control in several malignancies [2].

Natural products remain central to this field, not only for historical reasons but also because they continue to yield chemically distinctive and pharmacologically effective scaffolds. Plant metabolites have contributed substantially to the pharmacopeia of anticancer agents, as plant-derived compounds represent a significant share of approved drugs in oncology—a contribution rooted in the structural complexity and chemical diversity acquired through evolutionary selection, properties that confer a unique capacity to interact with biological macromolecules involved in tumor initiation, progression, and resistance [3]. Beyond their direct pharmacological utility, plant metabolites have also served as privileged scaffolds for semi-synthetic optimization, enabling compounds with improved potency, selectivity, and pharmacokinetic profiles relative to the original natural leads [3]. Several anticancer agents currently used in clinical practice exemplify this trajectory, including vincristine, vinblastine, paclitaxel, and camptothecin derivatives [3]. Their value lies in their ability to interfere with essential cellular processes such as proliferation, apoptosis, oxidative balance, and intracellular signaling [3]. Alkaloids are prominent plant secondary metabolites due to their structural breadth and biological versatility. Amaryllidaceae are particularly relevant in this regard. More than 600 alkaloids have been described in this family, with reports of acetylcholinesterase inhibition, antimicrobial activity, antioxidant effects, and cytotoxicity in tumor models [4]. This combination of chemical diversity and biological activity has kept this family in the focus of natural anticancer natural-product research [4,5].

The Brazilian genus *Griffinia* has been discussed from a less consolidated perspective. It comprises approximately 21 species, most of which are associated with the Atlantic Forest, Cerrado, and related ecosystems, and remains markedly underrepresented in phytochemical and pharmacological studies compared to other genera such as Narcissus, Crinum, and Zephyranthes [6,7]. However, the available evidence does not suggest that it is a chemically marginal genus. Reports based on gas chromatography–mass spectroscopy (GC–MS) and related analytical approaches have identified lycorine, galanthamine, crinine, hemanthamine, montanine, and homolycorine-type alkaloids in *Griffinia* species [7].

The strongest biological evidence currently available is that from *Griffinia gardneriana*; alkaloids isolated from bulbs, including lycorine, were shown to exhibit cytotoxic and genotoxic activities in experimental models and were associated with apoptosis involving caspase-3 activation [8]. These findings suggest that this genus is a pharmacological anchor. However, this does not justify the automatic extension of mechanistic claims to this genus.

This distinction is essential because lycorine and related Amaryllidaceae alkaloids have been examined in broader cancer-oriented literature, with reports on mitochondrial apoptosis, cell cycle arrest, and modulation of pathways such as NF-κB, JAK/STAT, and PI3K/Akt [9,10]. These data explain why *Griffinia* has attracted interest; however, this does not replace genus-specific evidence.

This review addresses a specific and unresolved gap in the Amaryllidaceae literature: despite the structural relevance of alkaloids detected in *Griffinia* and the growing interest in plant-derived anticancer scaffolds in the context of a disease projected to reach 35 million new cases globally by 2050 [1]. No prior review has systematically examined what the current evidence reports regarding the anticancer potential of this genus. The novelty of this work lies precisely in that distinction; it does not treat *Griffinia* as an established source of antitumor agents, but critically evaluates the phytochemical record, maps the existing biological evidence to specific species and compounds, and explicitly delineates the boundary between genus-level data and family-level extrapolations. By doing so, it establishes a transparent evidentiary framework for future pharmacological investigations of this underexplored Brazilian genus.

## 2. Literature Search Strategy

This narrative review was conducted in accordance with the scope and methodology appropriate for the phytochemical and pharmacological synthesis of an undercharacterized plant genus. A comprehensive literature search was performed across PubMed, Scopus, Web of Science, Google Scholar, SciFinder-n, and Reaxys, with no restrictions on publication date and limited to publications in English, Portuguese, or Spanish. SciFinder-n and Reaxys were additionally searched to improve the coverage of phytochemical reports, compound-level records, and structural information that may be underrepresented in biomedical databases. The primary search terms included “*Griffinia*”, “Amaryllidaceae alkaloids”, “lycorine anticancer”, “*Griffinia gardneriana*”, “phytochemistry Amaryllidaceae”, “cytotoxicity alkaloids”, and “apoptosis lycorine”. Boolean operators (AND, OR) were used to combine terms and refine search specificity. Studies were included if they reported (i) phytochemical identification or isolation of alkaloids from *Griffinia* species; (ii) biological or pharmacological evaluation of alkaloids reported in *Griffinia* or structurally related Amaryllidaceae compounds; or (iii) biosynthetic, taxonomic, or ecological data directly relevant to the genus. Studies were excluded if they addressed Amaryllidaceae genera with no structural overlap with alkaloid classes reported in *Griffinia* or if they presented pharmacological data without chemical characterization of the tested material. Family-level studies on Amaryllidaceae alkaloids were retained when the alkaloid class examined was demonstrably present in *Griffinia*, and these were explicitly distinguished from genus-specific evidence throughout the manuscript. The reference lists of the included papers were screened to identify additional relevant sources not captured by the primary database search. Following screening according to the predefined scope of the review, 47 references were retained in the final narrative synthesis. These publications comprised genus-specific studies addressing the taxonomy, phytochemistry, or biological properties of *Griffinia*, together with studies on Amaryllidaceae alkaloids relevant to the structural classes reported in the genus and complementary literature addressing anticancer mechanisms, natural-product drug discovery, biosynthesis, and methodological aspects. Notably, only a small subset of the retrieved literature provided direct phytochemical or pharmacological evidence for *Griffinia*, whereas most publications contributed family-level or compound-level contextual evidence. This marked imbalance between genus-specific primary evidence and the broader Amaryllidaceae literature was considered throughout the review and guided the distinction between direct evidence and mechanistic extrapolation.

## 3. Botanical and Phytochemical Overview of the Genus *Griffinia*

*Griffinia* is a taxonomically distinct genus of bulbous Amaryllidaceae endemic to Brazil, comprising approximately 21 species predominantly distributed across the Atlantic Forest, Cerrado, and adjacent transitional ecosystems [11]. Its morphological distinctiveness—marked by underground bulbs, pseudopetiolate leaves with reticulate venation, and zygomorphic flowers with blue-to-violet pigmentation—is not merely of descriptive value: these traits underpin the taxonomic circumscription of the genus within Amaryllidaceae and reflect evolutionary adaptations associated with specific ecological niches, pollination strategies, and soil-mediated biochemical pressures that are known to influence the composition and abundance of secondary metabolites in bulbous plants [11,12]. The geographic confinement of most species to biodiversity-rich and environmentally heterogeneous regions of Brazil is therefore directly relevant to the phytochemical discussion: habitat specialization and restricted distribution create conditions conducive to metabolite diversification, and the high species richness concentrated in the Atlantic Forest and Cerrado suggests that the alkaloid diversity currently documented for *Griffinia* likely represents only a fraction of the chemical potential of the genus. A representative specimen of *Griffinia liboniana* Morren cultivated under controlled conditions is shown in Figure 1 to illustrate the morphological features discussed. Despite its taxonomic distinctiveness and ecological relevance, the genus remains substantially underexplored from phytochemical and pharmacological perspectives—a gap that this review directly addresses.

The geographic distribution of *Griffinia* species in Brazil is heterogeneous and closely associated with biodiversity-rich regions, particularly the Atlantic Forest and transitional ecosystems. This spatial distribution is relevant from a taxonomic perspective and for understanding the patterns of phytochemical diversity, species accessibility, and conservation status.

Figure 2 illustrates the occurrence and relative richness of *Griffinia* species across Brazilian states, highlighting regions with higher species concentrations. This distribution pattern suggests that areas with high species richness may be priority targets for phytochemical screening and pharmacological investigations, as local environmental conditions can influence metabolite diversity and abundance. The restricted distribution of several species reinforces the importance of sustainable exploration and conservation-oriented research strategies, particularly in ecologically vulnerable regions.

The geographical heterogeneity of *Griffinia* raises a relevant, although still unresolved, chemotaxonomic question. Species occurring across the Atlantic Forest, Cerrado, and transitional environments are exposed to distinct ecological pressures that may contribute to variation in specialized metabolism. From a biosynthetic perspective, the lycorine-, galanthamine-, crinine-, hemanthamine-, montanine-, and homolycorine-type alkaloids reported in the genus derive from the shared Amaryllidaceae isoquinoline alkaloid biosynthetic framework. However, their distribution cannot currently be reliably associated with particular habitats or evolutionary lineages within *Griffinia*, because phytochemical characterization remains restricted to a small proportion of the genus and differs substantially in analytical depth among species. Consequently, the apparent occurrence of different alkaloid skeletons should not yet be interpreted as evidence of habitat-driven chemical differentiation or phylogenetic clustering. Integrating species-resolved phylogenetic analyses with standardized metabolomic profiling and georeferenced sampling represents an important future approach for determining whether evolutionary history and environmental heterogeneity contribute to alkaloid diversification in *Griffinia*.

The concentrations of species in southeastern and northeastern Brazil suggest that these are priority regions for future phytochemical and pharmacological investigations. In this broader context, the family Amaryllidaceae provides an essential chemical and biological framework. This family is one of the most extensively studied in alkaloid research and is characterized by a large and structurally diverse repertoire of specialized metabolites associated with multiple biological activities, including cholinesterase inhibition, antimicrobial effects, antioxidant responses, and tumor cell cytotoxicity [4,15,16].

This family background establishes the pharmacognostic relevance of *Griffinia* from the outset; however, it raises the evidentiary threshold, as the mere detection of family-typical alkaloids is insufficient to support pharmacological generalization. Accordingly, the phytochemical record available for *Griffinia* remains uneven, although informative. Gas chromatography–mass spectrometry (GC–MS) and liquid chromatography (LC) (LC–MS) have been used to identify the alkaloid class characteristics of this family, including lycorine, galanthamine, crinine, hemanthamine, montanine, and homolycorine-type compounds [17]. These findings place *Griffinia* within the alkaloid chemistry of Amaryllidaceae; however, the degree of phytochemical characterization varies substantially across species, and chemical detection alone does not translate directly into pharmacological understanding. As documented for other genera of this family, the biological relevance of individual alkaloid classes depends not only on their structural identity but also on concentration, matrix composition, and the specific assay conditions under which activity is evaluated—factors that remain largely unaddressed for most *Griffinia* species. Moreover, the toxicological dimension must be considered alongside pharmacological interest; since several Amaryllidaceae alkaloids exhibit cytotoxic effects that are not inherently selective for tumor cells, a distinction that becomes critical when translational potential is discussed [18]. Among the taxa examined thus far, *G. gardneriana* stands out as the only species for which phytochemical investigation has progressed beyond analytical profiling to the isolation of individual alkaloids and subsequent biological evaluation [8,18,19]. This level of characterization distinguishes it from other *Griffinia* species, for which the available evidence remains predominantly based on chromatographic or spectrometric detection. Nevertheless, differences among the genera remain pronounced. An integrative assessment of Amaryllidaceae occurring in the Cerrado, a biome that hosts *Griffinia* alongside *Crinum, Hippeastrum, Nothoscordum*, and *Zephyranthes*, found that chemical composition or biological activity data are entirely absent for approximately 70% of the evaluated species, with *Griffinia* among the genera for which this deficiency is most acute [20]. Although some species have been investigated through chemical identification and biological evaluation, others are represented only by class-level assignments or preliminary phytochemical reports. This asymmetry is explicitly reflected in Table 1: the uneven depth of evidence is not a secondary detail but a defining characteristic of the current knowledge base on *Griffinia*, and it mirrors a broader pattern of underinvestigation that extends across much of the Amaryllidaceae of the Brazilian Cerrado [20].

## 4. Chemical Diversity of Alkaloids in the Genus *Griffinia*: Phytochemical Evidence and Pharmacological Gaps

The alkaloid profile reported for *Griffinia* is part of a broader biosynthetic framework characteristic of Amaryllidaceae. In this family, isoquinoline-derived alkaloids arise from O-methylnorbelladine via intramolecular oxidative phenolic coupling, generating the main structural skeletons described in the literature [21,22]. This shared biosynthetic origin explains the structural diversity of alkaloid types found across related genera and provides a chemical rationale for the presence of comparable compound classes in *Griffinia*. However, a critical distinction must be established at the outset of this section: the identification of an alkaloid class through chromatographic or spectrometric profiling, the predominant form of evidence available for *Griffinia*, establishes chemical occurrence but does not constitute pharmacological characterization. Knowing that a compound is present in a plant extract is not equivalent to demonstrating its biological activity, potency, selectivity, or mechanism of action under defined experimental conditions. For most species of *Griffinia*, the available evidence does not extend beyond this first level, and the depth of chemical characterization varies substantially across the genus. This asymmetry between identification and validation is not a minor technical detail; it defines the interpretative boundaries of the entire phytochemical discussion that follows. The alkaloid profile reported for *Griffinia* is part of a broader biosynthetic framework characteristic of Amaryllidaceae. In this family, isoquinoline-derived alkaloids arise from O-methylnorbelladine via intramolecular oxidative phenolic coupling, generating the main structural skeletons described in the literature [21,22]. This shared origin explains the diversity of alkaloid types found in these related genera.

Within *Griffinia*, the chemical record is broader than that of a single dominant class. The reports for this genus include lycorine, galanthamine, crinine, hemanthamine, montanine, and homolycorine-type alkaloids [7,19,23]. This range is relevant, indicating that the genus is chemically integrated into the major structural repertoire of Amaryllidaceae, rather than occupying an isolated or marginal position.

Lycorine-type compounds are the most prominent in the literature. This recurrence is not surprising, since lycorine is one of the best-studied alkaloids in Amaryllidaceae research, especially in studies involving tumor cell proliferation, apoptosis, oxidative stress, mitochondrial dysfunction, and cell cycle regulation [23,24]. However, in the case of *Griffinia*, the importance of lycorine begins with its occurrence and not with a fully developed mechanism-based profile.

The presence of galanthamine-type alkaloids has broadened the discussion in other directions. These compounds are more commonly associated with acetylcholinesterase inhibition and the therapeutic use of galanthamine in Alzheimer’s disease [20,25], suggesting that the chemistry of *Griffinia* is not restricted to classes usually emphasized in oncology. This is true for crinine-, hemanthamine-, montanine-, and homolycorine-type alkaloids. They expand the chemical landscape of the genus, even though biological characterization remains limited.

The predominant gap is not chemical diversity, which is already demonstrable, but the depth of characterization, particularly the progression from chromatographic detection to compound isolation and structural elucidation. GC–MS and LC–MS studies have identified molecules such as lycorine, vittatine, and sanguinine in *Griffinia* species [7,8]. This is sufficient to demonstrate their chemical richness and justify continued interest. However, it is insufficient to define the pharmacological behavior of the genus with the same level of confidence. Much of the literature stops at identification, whereas metabolomic approaches and structure–activity analyses remain limited.

Therefore, the main result of this section is not dramatic but solid: *Griffinia* has a diversified alkaloid profile and belongs, without doubt, to the chemical conversation of Amaryllidaceae. However, the depth of this research is still lacking.

Table 2 summarizes this distinction and additionally classifies the structural evidence supporting the reported alkaloids. The evidence levels distinguish compounds confirmed after isolation and structural characterization from putative GC–MS/LC–MS assignments and preliminary class-level reports. This classification is essential because chromatographic or mass-spectrometric detection alone should not be interpreted as equivalent to complete structural confirmation.

## 5. Anticancer-Relevant Evidence for Alkaloids Reported in the Genus *Griffinia*

### 5.1. Direct Experimental Evidence from Griffinia gardneriana

Direct pharmacological evidence within *Griffinia* is currently restricted to *G. gardneriana*. In the study by Cole et al. [8], alkaloid-enriched fractions obtained from the bulbs were evaluated by the MTT assay against OVCAR-3, J774, and L929 cells. The n-hexane, ethyl acetate, and EtOAc/MeOH (3:1) fractions showed IC_50_ values of 31.31, 38.02, and 45.52 µg/mL, respectively, against OVCAR-3 cells, while IC_50_ values against J774 were 12.40 µg/mL for all three fractions and those against L929 ranged from 12.40 to 14.30 µg/mL. These results indicate cytotoxic activity of the enriched fractions but do not, by themselves, establish tumor selectivity.

Among the isolated alkaloids associated with *G. gardneriana*, lycorine and pretazettine displayed the most pronounced cytotoxic effects. After 48 h of exposure, lycorine showed an IC_50_ of 28.89 ± 7.59 µM against MCF-7 cells, whereas pretazettine showed an IC_50_ of 38.02 ± 8.41 µM. Neither compound reached an IC_50_ against HepG2 cells within the tested concentration range (>174.02 µM for lycorine and >150.89 µM for pretazettine). Importantly, stronger cytotoxicity was observed after 72 h: lycorine exhibited IC_50_ values of 1.15 ± 0.49 µM in A549 and 4.38 ± 0.80 µM in MCF-7 cells, whereas pretazettine yielded IC_50_ values of 4.95 ± 0.51 and 7.03 ± 0.99 µM, respectively. These findings demonstrate a marked time-dependent increase in cytotoxic activity. However, activity against non-tumor L929 fibroblasts was also observed, with 48 h IC_50_ values of 8.80 ± 4.20 µM for lycorine and 0.78 ± 2.93 µM for pretazettine, underscoring the need for cautious interpretation of tumor selectivity [8].

Genotoxicity was additionally evaluated in CHO-1-15 cells using the cytokinesis-block micronucleus assay. At the concentrations selected from the A549 cytotoxicity experiments, lycorine (1.15 ± 0.49 µM) produced micronuclei, nuclear buds, and nucleoplasmic bridges at frequencies of 7.50 ± 3.54%, 5.00 ± 2.83%, and 8.50 ± 0.71%, respectively, with a reported relative cytotoxicity of 63.68%. Pretazettine (4.95 ± 0.51 µM) produced corresponding frequencies of 10.00 ± 1.73%, 7.33 ± 1.15%, and 4.67 ± 1.00%, with a relative cytotoxicity of 49.10%. Both compounds were also evaluated in A549 cells for caspase-3-associated apoptosis, supporting activation of this apoptotic pathway at the concentrations tested [8]. Together, these quantitative findings constitute the most detailed direct pharmacological evidence currently available for the genus. Nevertheless, the concomitant activity in non-tumor cells and the genotoxic responses indicate that cytotoxic potency should not be equated with selective anticancer efficacy.

### 5.2. Contextual Anticancer Evidence from Structurally Related Amaryllidaceae Alkaloids

Beyond the direct evidence from *G. gardneriana*, a substantially larger body of literature describes anticancer effects of lycorine and other structurally related Amaryllidaceae alkaloids investigated outside *Griffinia.* These studies provide mechanistic and pharmacological context for evaluating the biological plausibility of alkaloid classes detected in the genus; however, they constitute indirect evidence and should not be interpreted as demonstrating anticancer activity of *Griffinia* species. Lycorine occupies a central position in this debate because it links direct reports on *Griffinia* to a broader body of research on alkaloids in Amaryllidaceae. In leukemia, hepatocellular carcinoma, breast cancer, and lung cancer models, lycorine has been associated with reduced proliferation, inhibition of protein synthesis, mitochondrial apoptosis, and modulation of oxidative stress-related processes [10,22,25]. Although these reports do not provide direct evidence of the behavior of *Griffinia* extracts or species, they strengthen the biological plausibility for further investigation of lycorine-type compounds in this genus.

Apoptosis is a well-established phenomenon reported in the literature. In studies on lycorine and related compounds, intrinsic apoptotic signaling has been linked to mitochondrial dysfunction, cytochrome c release, and the activation of caspase cascades, particularly caspase-3 and caspase-9 [25]. The observation of caspase-3-associated apoptosis in *G. gardneriana* aligns with a known pharmacological pattern rather than being an isolated finding.

Other anticancer processes are more clearly elucidated from family-level studies than from genus-specific studies. Cell cycle arrest is an example, with reports outside *Griffinia* describing interference with the G0/G1 and G2/M checkpoints and changes in proteins involved in cell cycle regulation [26]. Oxidative stress is another factor. Reactive oxygen species (ROS) may either sustain tumor adaptation or contribute to mitochondrial injury and cell death, with several Amaryllidaceae alkaloids linked to altered ROS dynamics in tumor cells [25,26,27]. The mechanistic and molecular activity of alkaloids from the Amaryllidaceae family extends beyond the classical pathways of apoptosis induction and cell-cycle regulation discussed previously. These compounds have demonstrated cholinesterase inhibitory, antiviral, and antitumor activities, indicating a broad pharmacological profile associated with their structural diversity [27,28]. Mechanistic studies suggest that this antitumor activity involves modulation of multiple cellular signaling pathways. In particular, lycorine and structurally related alkaloids have shown cytotoxic effects in different cancer models, including cells resistant to apoptotic stimuli, while structure–activity relationship studies indicate that the phenanthridine nucleus contributes significantly to this biological activity [29,30].

Lycorine is among the most studied Amaryllidaceae alkaloids and has demonstrated antiproliferative effects in several experimental tumor models, supporting its relevance as a reference compound for understanding the pharmacological potential of alkaloid classes identified in *Griffinia* [31]. In addition, semi-synthetic studies involving norbelladine-derived precursors have contributed to the investigation of structural features associated with biological activity within this alkaloid family [32].

Evidence also indicates that lycorine may interfere with cellular metabolism through mechanisms involving IDH1 acetylation and alterations in mitochondrial dynamics in colorectal cancer cells [33]. ROS modulation represents another relevant mechanism, since oxidative stress may influence tumor progression and therapeutic response [34,35].

At the molecular level, lycorine has been reported to modulate signaling pathways such as Wnt/β-catenin, ERK1/2-MAPK, and PI3K/AKT in osteosarcoma models [36], as well as the FBXW7-MCL1 axis in gastric cancer cells [37]. Other studies involving Amaryllidaceae alkaloids have also described effects on cytoskeletal organization, matrix metalloproteinase expression, and inflammatory cytokine production in breast and colon cancer models [38,39]. Together, these findings support the pharmacological relevance of the alkaloid classes detected in *Griffinia*. However, direct experimental studies with this genus remain necessary to confirm its specific biological effects and toxicological profile.

Accordingly, Figure 3 and Table 2 should be interpreted as contextual syntheses of biological processes associated with alkaloid classes reported in *Griffinia*, rather than as evidence that these mechanisms have been experimentally demonstrated across the genus.

## 6. Molecular Mechanisms Relevant to the Anticancer Activity of Amaryllidaceae Alkaloids

Before discussing the molecular mechanisms associated with Amaryllidaceae alkaloids relevant to the phytochemical profile of *Griffinia*, an important evidentiary distinction must be emphasized. With the exception of the limited apoptosis-related evidence reported for *G. gardneriana*, the molecular pathways discussed in this section have not been experimentally demonstrated in *Griffinia* species. Evidence involving PI3K/AKT, MAPK, NF-κB, Wnt/β-catenin, mitochondrial signaling, and related molecular targets derives predominantly from studies of isolated Amaryllidaceae alkaloids investigated in experimental systems outside the genus. These findings are therefore presented as mechanistic context for alkaloid classes chemically reported in *Griffinia* and should not be interpreted as demonstrated molecular mechanisms of the genus.

Within this broader Amaryllidaceae literature, lycorine is among the most extensively investigated alkaloids. Apoptosis is one of the most consistently reported outcomes, and in several tumor models lycorine has been associated with loss of mitochondrial membrane potential, cytochrome c release, and activation of the caspase cascade [26,27,29]. This recurrent association contributes to the continued interest in lycorine and structurally related Amaryllidaceae alkaloids in cancer pharmacology.

The second major mechanistic pathway is cell cycle regulation. Several studies have reported arrest at the G0/G1 or G2/M phase, accompanied by changes in cyclins, cyclin-dependent kinases, and other regulators of proliferative control [26,30,31,32]. These observations broaden the interpretation of Amaryllidaceae alkaloids beyond their crude cytotoxicity and place them within a more defined antiproliferative framework.

Redox imbalance has been reported repeatedly in the literature. ROS play context-dependent roles in cancer biology, and some Amaryllidaceae alkaloids have been associated with altered intracellular ROS levels in tumor cells [25,26]. Under certain conditions, this shift may contribute to mitochondrial stress and activate cell death pathways. Under other conditions, biological significance is less straightforward. Therefore, ROS-related findings should be considered mechanistically relevant but not self-sufficient.

In addition to apoptosis and cell cycle effects, several studies have examined the signaling pathways involved in tumor maintenance and progression. Lycorine and related compounds have been associated with the modulation of PI3K/Akt, NF-κB, Wnt/β-catenin, and MAPK pathways [22,33,37]. In some models, these alkaloids have been linked to reduced cell migration, invasion, and tumor-associated cytokine secretion [38,39]. This considerably widens their pharmacological profiles, although the depth of evidence varies among pathways. To provide a more detailed molecular-level synthesis, the experimentally reported molecular targets and signaling components associated with the Amaryllidaceae alkaloids discussed in this review are summarized in Supplementary Table S1. The table organizes the available evidence according to alkaloid, experimental model, molecular target or signaling component, associated pathway or biological process, biological outcome, and its relationship to the evidence available for *Griffinia*. Most of these molecular associations derive from studies conducted with isolated Amaryllidaceae alkaloids outside the genus and should therefore be interpreted as mechanistic context rather than as demonstrated molecular targets of *Griffinia* compounds.

However, direct mechanistic evidence for *Griffinia* remains limited. The clearest case is that of *G. gardneriana*, in which isolated alkaloids, including lycorine, showed cytotoxic and genotoxic activities associated with apoptosis involving caspase-3 activation [19]. At present, this is a real mechanistic anchor, but it is not a sufficient basis for a genus-wide mechanism map.

Therefore, Table 3 should be maintained as a comparative synthesis of the mechanisms described for selected Amaryllidaceae alkaloids relevant to the structural classes reported in *Griffinia*. This distinction is important. It preserves the mechanistic value of the broader family literature without converting it into unsupported proof of the genus.

### Toxicological Considerations and Translational Limitations

Toxicological characterization represents an additional limitation in assessing the translational potential of *Griffinia* alkaloids. Within the genus, the available evidence is largely restricted to the genotoxic effects reported for alkaloids isolated from *G. gardneriana* [8], whereas systematic data on neurotoxicity, hepatotoxicity, nephrotoxicity, and systemic tolerability remain unavailable. Therefore, toxicity profiles described for other Amaryllidaceae taxa or isolated alkaloids should not be directly attributed to *Griffinia* [18].

At the family level, however, toxicological concerns associated with Amaryllidaceae alkaloids have been documented. Exposure to Amaryllidaceae plants and their alkaloids has been associated with gastrointestinal manifestations, including nausea, vomiting, diarrhea, and abdominal discomfort, as well as neurological and cardiovascular effects, depending on the species, alkaloid composition, dose, and exposure conditions [18]. Available reports also indicate that hepatic and renal effects should be considered in the safety assessment of these compounds, although the evidence remains heterogeneous and cannot be generalized across individual alkaloids or taxa [18]. These observations reinforce the need to distinguish pharmacological activity from an acceptable therapeutic window, particularly when these alkaloids are considered as potential anticancer leads [18,40].

From a translational perspective, cytotoxic potency alone is insufficient to support progression toward anticancer development. Candidate alkaloids require systematic determination of tumor-cell selectivity, concentration–response relationships in non-tumor cells, acute and repeated-dose toxicity, and organ-specific effects. Pharmacokinetic characterization is also essential to determine whether biologically active concentrations can be achieved in vivo without unacceptable systemic exposure [40,41,42]. For *Griffinia*, these requirements remain largely unmet and represent a major bottleneck between preliminary cytotoxic observations and preclinical development. Consequently, future studies should integrate efficacy and safety assessment from the early stages of pharmacological characterization rather than treating toxicity as a secondary endpoint.

## 7. Challenges and Future Perspectives in the Development of Anticancer Agents from *Griffinia* Alkaloids

The transition from phytochemical discovery to clinically relevant anticancer agents follows a structured progression: compound identification and characterization, biological validation, pharmacological optimization, and preclinical-to-clinical translation. In *Griffinia*, this progression remains stalled at the early stages. The main limitation is not the absence of chemical interest but the weak continuity between phytochemical detection and pharmacological validation, a gap that mirrors broader patterns documented across alkaloid-producing genera in oncological drug discovery [40,41]. For most species, chemical reporting has advanced faster than biological testing; therefore, the genus should be regarded as chemically promising but biologically undercharacterized—a starting point for systematic investigation, not a source of established drug candidates.

Three specific and hierarchically ordered obstacles define the current research agenda. The first is compound availability. Many alkaloids in Amaryllidaceae occur in low abundance, and in *Griffinia*, incomplete phytochemical coverage and restricted access to plant materials intensify this problem [20]. Under these conditions, plant tissue culture, metabolic engineering, and heterologous biosynthesis are not peripheral options but plausible and increasingly explored strategies for overcoming supply bottlenecks [43,44]. Resolving this obstacle is a prerequisite for all subsequent steps: pharmacological studies cannot advance without reproducible access to structurally defined, genus-specific compounds.

The second obstacle is pharmacological optimization. Natural alkaloids may exhibit cytotoxic or antiproliferative effects but are frequently constrained by toxicity, poor selectivity, limited stability, and inadequate bioavailability [18]. Semi-synthetic modifications can improve pharmacokinetic behavior and selectivity without discarding the original scaffold [45,46], and structure–activity relationship elucidation is the essential prior step for guiding rational optimization and identifying lead candidates suitable for further development [26]. For *Griffinia*, however, this stage remains largely inaccessible because the field still lacks a direct comparative assessment of genus-specific compounds, and standardized protocols for extraction, isolation, and structural characterization have not been established across species.

The third and most analytically demanding obstacle is mechanistic resolution. Pathway-level evidence for Amaryllidaceae alkaloids, covering mitochondrial apoptosis, cell-cycle arrest, and modulation of PI3K/Akt, NF-κB, and MAPK signaling, has been discussed in preceding sections; however, detailed activity–mechanism studies remain unavailable for most compounds, and genus-specific evidence is especially scarce. Mechanistic investigations must advance beyond indirect inferences based on structural similarity and rely on direct experimental validation using compounds isolated from *Griffinia* species. Transcriptomic, proteomic, metabolomic, and integrated multi-omic approaches are particularly well-suited to this challenge, as they can clarify the biological networks affected and distinguish primary pharmacological effects from secondary or adaptive responses [47,48]. Before combination strategies or formulation approaches can be meaningfully pursued for *Griffinia* alkaloids, three sequential priorities must be addressed: (i) isolation and full structural elucidation of genus-specific compounds using NMR-based methods; (ii) direct bioactivity screening against well-characterized cancer cell lines to establish potency, selectivity, and spectrum of activity; and (iii) mechanistic investigation anchored in genus-specific experimental data rather than structural inferences. A targeted experimental roadmap for *Griffinia* should therefore proceed in sequential stages. First, ex situ propagation and tissue-culture systems using bulb-derived explants should be explored to provide reproducible plant material while reducing pressure on naturally restricted populations. These platforms could subsequently support controlled studies of alkaloid production under standardized conditions. Second, phytochemical investigation should prioritize the recovery of low-abundance alkaloids through optimized extraction and fractionation, followed by high-resolution chromatographic separation and complete structure elucidation using MS- and NMR-based approaches. Third, structurally confirmed compounds should undergo comparative in vitro screening in well-characterized cancer and non-tumor cell models to establish potency and selectivity before extensive mechanistic investigation. Compounds displaying reproducible activity and an acceptable selectivity profile could then progress to appropriate in vivo tumor models for preliminary assessment of antitumor efficacy, systemic toxicity, pharmacokinetic behavior, and exposure–response relationships. This stepwise strategy would establish a more rigorous progression from phytochemical discovery to pharmacological validation while avoiding premature translational claims. These prerequisites are not peripheral but constitute the necessary foundation for subsequent pharmacological development.

Accordingly, progression from predominantly in vitro evidence to appropriately designed preclinical studies represents a critical next step for the field [20,40,41]. Combination strategies with established chemotherapeutics may eventually reveal synergy or permit dose reduction [42,49]; however, this line of investigation should not precede the basic pharmacological characterization that *Griffinia* has yet to receive.

## 8. Limitations

This review has limitations inherent to both its narrative design and the restricted primary literature available for *Griffinia*. The narrative synthesis may introduce selection bias, while the small number of phytochemically investigated species limits genus-wide generalization. In addition, most reported alkaloids have been assigned primarily by GC–MS or LC–MS, frequently without isolation and NMR-based structural confirmation, and direct pharmacological evidence remains largely restricted to *G. gardneriana*. These constraints were considered throughout the review by explicitly distinguishing genus-specific findings from biological evidence derived from other Amaryllidaceae taxa.

## 9. Conclusions

The available literature establishes *Griffinia* as a phytochemically relevant but pharmacologically undercharacterized genus within Amaryllidaceae. Lycorine-, galanthamine-, crinine-, hemanthamine-, montanine-, and homolycorine-type alkaloids have been reported across the genus, demonstrating substantial chemical diversity. However, direct pharmacological evidence remains concentrated in *G. gardneriana*, whose bulb alkaloids, including lycorine, have shown cytotoxic and genotoxic effects associated with caspase-3-mediated apoptosis. Thus, the current evidence supports the chemical relevance and biological plausibility of *Griffinia*, but not a genus-wide anticancer profile.

Evidence from structurally related Amaryllidaceae alkaloids provides a valuable mechanistic framework for prioritizing future investigations but should not be interpreted as direct evidence for *Griffinia*. Progress toward anticancer drug discovery will require species-specific experimental validation, particularly regarding pharmacological selectivity, mechanism of action, and in vivo activity. At present, *Griffinia* should therefore be regarded not as an established source of antitumor agents, but as an underexplored genus with a sufficiently coherent phytochemical and preliminary pharmacological basis to justify systematic investigation.

## Figures and Tables

**Figure 1:**
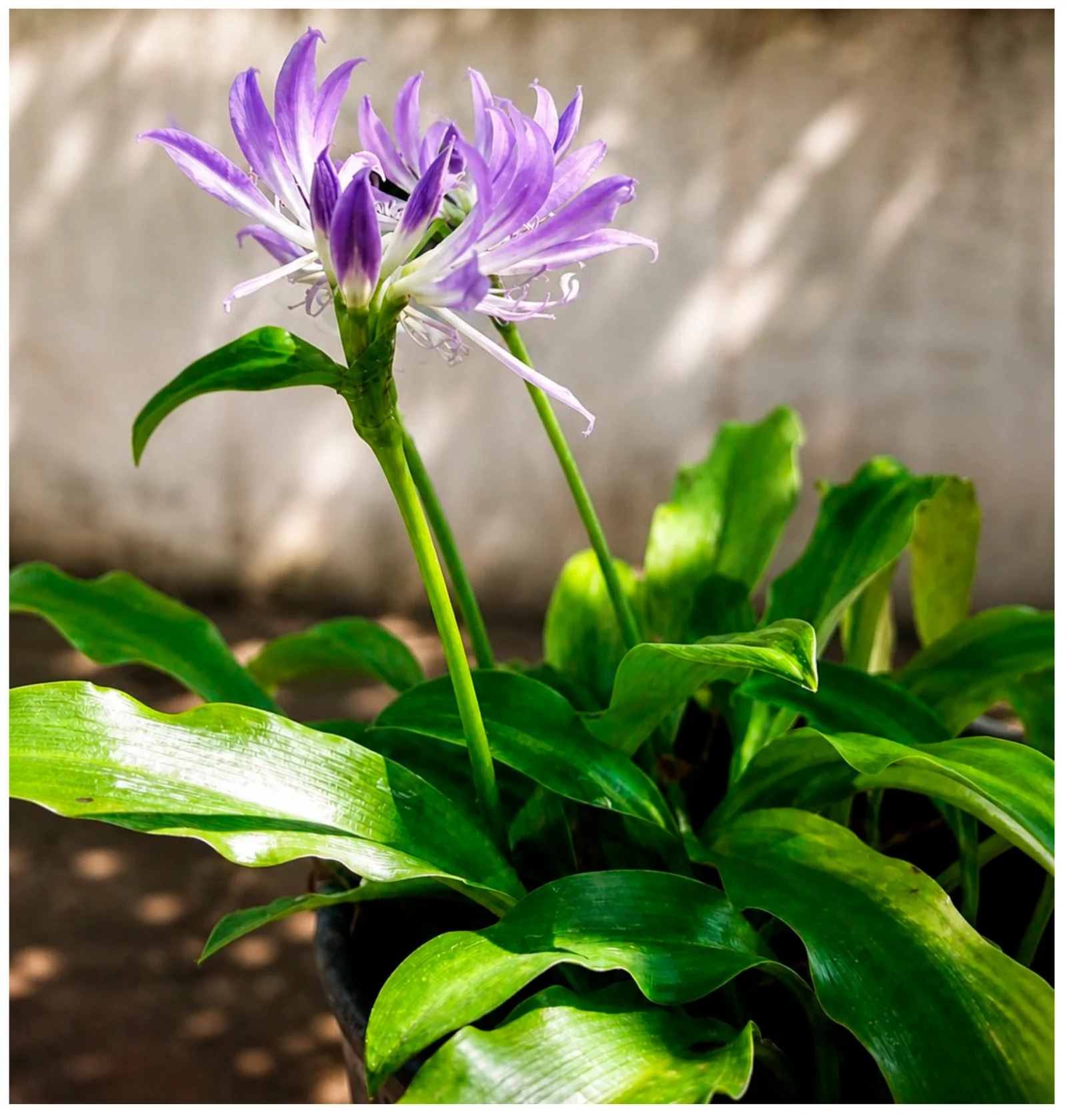
Morphological characteristics of *Griffinia liboniana* Morren (Amaryllidaceae). The image highlights the key botanical features of the species, including elongated leaves with reticulate venation and a zygomorphic flower with characteristic violet pigmentation. These traits support taxonomic identification and provide a morphological context for the phytochemical and pharmacological discussions presented in this review. Source: Author’s archive.

**Figure 2:**
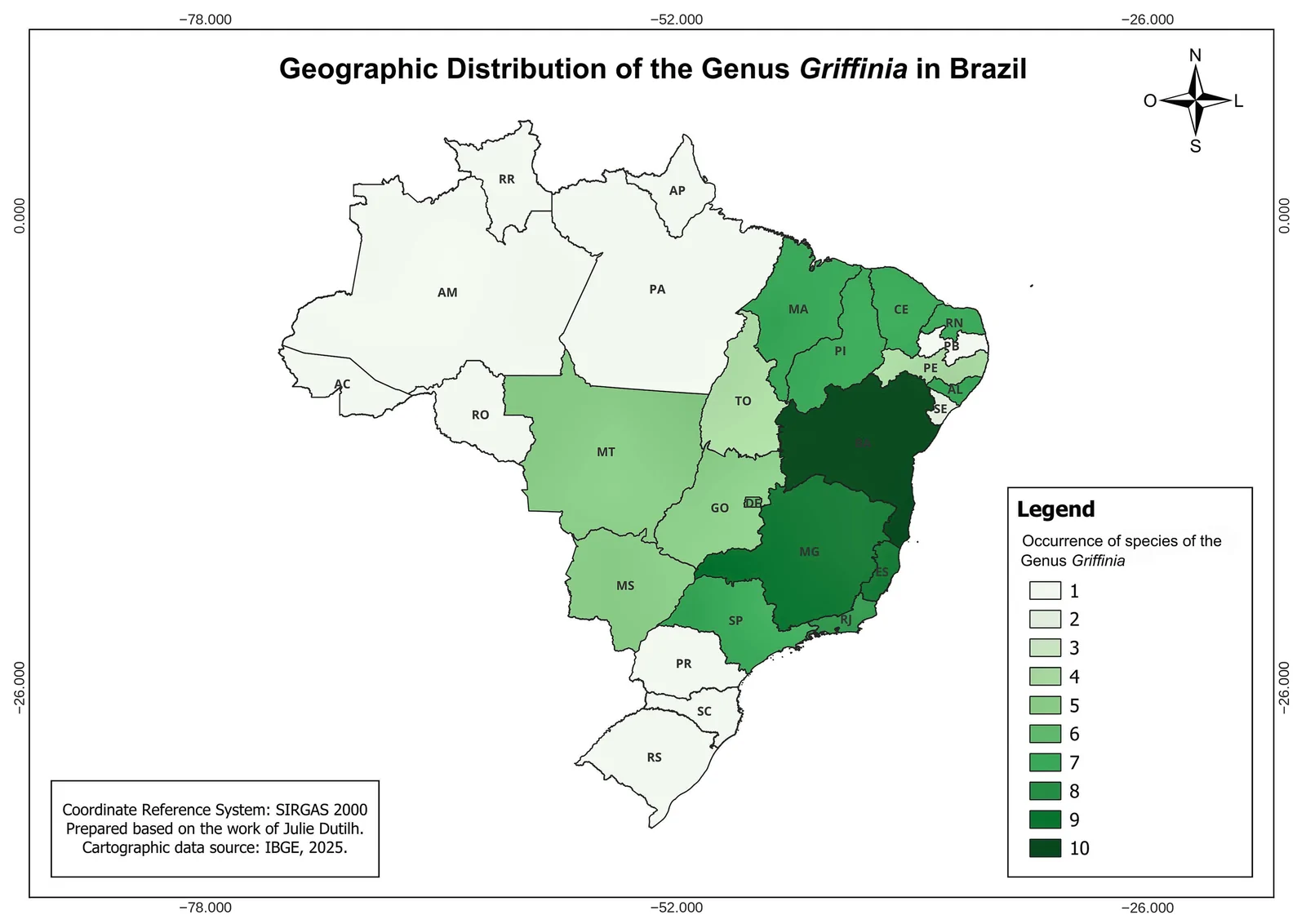
Geographic distribution of the genus *Griffinia* in Brazil. The map illustrates the occurrence and relative richness of *Griffinia* species across Brazilian states based on occurrence records compiled from the Flora e Funga do Brasil database (Jardim Botânico do Rio de Janeiro, available at: https://floradobrasil.jbrj.gov.br, accessed on 1 May 2025) and the Global Biodiversity Information Facility (GBIF; available at: https://www.gbif.org, accessed on 1 May 2025) [13]. Darker shades indicate regions with higher species representation, particularly in the Atlantic Forest and adjacent areas. This spatial pattern provides an ecological context for the phytochemical and pharmacological relevance of this genus. Coordinate reference system: SIRGAS 2000. Cartographic base (state boundaries): IBGE (2025) [14].

**Figure 3:**
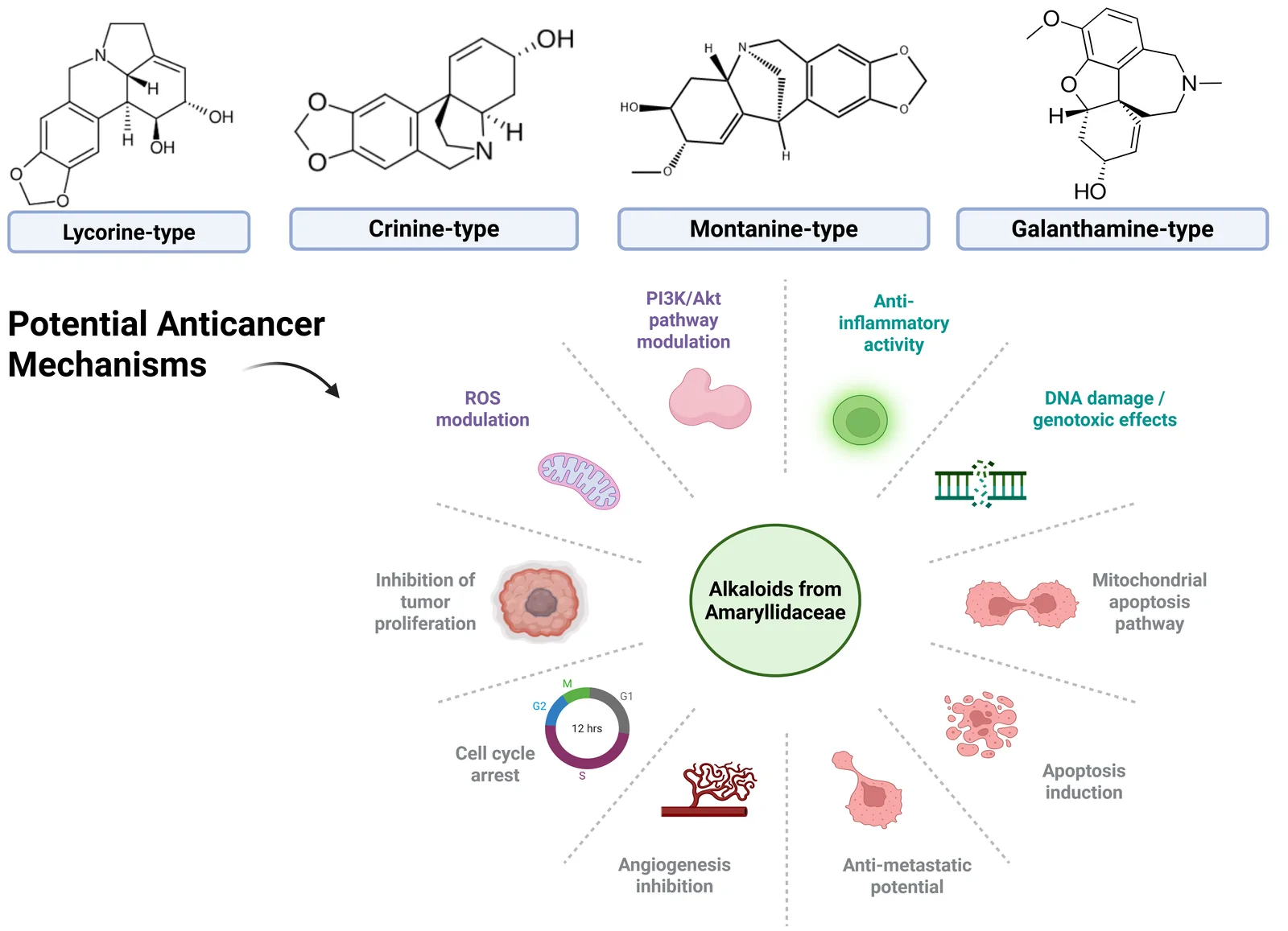
Representative structural classes of Amaryllidaceae alkaloids and their proposed anticancer mechanisms. The upper panel illustrates the selected alkaloid skeletons commonly reported in Amaryllidaceae, including lycorine, crinine, montanine, and galanthamine-type structures. The lower schematic summarizes the main biological processes associated with these compounds based on experimental evidence from the literature, including modulation of ROS, inhibition of tumor cell proliferation, induction of apoptosis (including mitochondrial pathways), cell cycle arrest, anti-inflammatory activity, DNA damage/genotoxic effects, inhibition of angiogenesis, and anti-metastatic potential. These mechanisms are primarily derived from studies on well-characterized Amaryllidaceae alkaloids and should be interpreted with caution when extrapolated to underexplored genera such as *Griffinia*. Created in BioRender. Jhuly, M. (2026) https://BioRender.com/rv0yyj4.

**Table 1:** Selected species of the genus *Griffinia* and current phytochemical evidence regarding reported alkaloids.

Species	Geographic Distribution	Plant Part	Reported Alkaloid Classes/Compounds	Level of Phytochemical Characterization	References
*Griffinia gardneriana*	Brazil (Atlantic Forest)	Bulbs	Lycorine-type	Isolated compound with full biological evaluation (cytotoxicity, genotoxicity, apoptosis assay)	[17,19]
*Griffinia liboniana*	Brazil	Bulbs	Lycorine-type and galanthamine-type	Class-level assignment by GC–MS profiling; no isolated pure compound reported	[17]
*Griffinia nocturna*	Brazil	Bulbs	Crinine-type, homolycorine-type	Class-level assignment by GC–MS profiling; no isolated pure compound reported	[17]
*Griffinia espiritensis*	Brazil	Not specified (NS)	Amaryllidaceae alkaloids reported at genus level; specific class or compound not specified in the primary source	Preliminary phytochemical indication only; insufficient data for class-level characterization	[11]

**Table 2:** Major alkaloid classes reported in the genus *Griffinia*: direct evidence in *Griffinia* and biological activities described for related Amaryllidaceae alkaloids.

Alkaloid Class	Representative Compounds	Biological Activities	Structural Evidence Level	References
Lycorine-type	Lycorine (isolated, *G. gardneriana*); vittatine and sanguinine (detected by GC–MS)	Cytotoxic, antiproliferative, apoptosis induction, cell-cycle modulation	Level I for isolated lycorine; Level II for GC–MS assignments	[7,8,10,23,24]
Galanthamine-type	Galanthamine-type alkaloids	Acetylcholinesterase inhibition; neuroprotective activity	Level II—putative/class-level assignment by GC–MS	[15]
Crinine-type	Crinine-type alkaloids	Cytotoxic and antiproliferative effects described in the family	Level II—putative/class-level assignment by GC–MS	[23]
Haemanthamine-type	Hemanthamine	Cytotoxic and antiproliferative effects described in the family	Level II—putative assignment; nuclear resonance magnetic (NMR) confirmation required	[23]
Homolycorine-type	Homolycorine-type alkaloids	Anti-inflammatory and antimicrobial activities reported in the family	Level II—putative/class-level assignment by GC–MS	[15]
Montanine-type	Montanine-type alkaloids	Antiproliferative activity described in in vitro models	Level II/III—class-level evidence; discrete compound identity not confirmed	[20]

Evidence levels: Level I (confirmed), isolated compound with structural characterization supported by spectroscopic methods, including NMR where reported; Level II (putatively identified), compound or alkaloid class assigned primarily by GC–MS or LC–MS based on chromatographic and/or mass-spectral evidence without complete NMR confirmation; Level III (preliminary), alkaloid occurrence reported at the class or genus level without sufficient analytical evidence for confident compound-level identification. Evidence levels refer exclusively to the confidence of chemical identification and should not be interpreted as levels of pharmacological evidence.

**Table 3:** Molecular mechanisms relevant to the anticancer activity of selected Amaryllidaceae alkaloids discussed in this review.

Alkaloid	Source/Structural Class	Cancer Model	Molecular Mechanism Relevant to Anticancer Activity	References
Lycorine	Lycorine-type alkaloid	Hepatocellular carcinoma (HepG2)	Induction of mitochondrial apoptosis; activation of caspase-3 and caspase-9	[25,34]
Lycorine	Lycorine-type alkaloid	Leukemia cells	Cell-cycle arrest; inhibition of eukaryotic protein synthesis via 60S ribosomal subunit interference	[22,34]
Pretazettine	Lycorine-type alkaloid sensu lato; structural placement within the lycorine series is based on the isoquinoline *C*-ring system shared with lycorine-type compounds, although its precise skeletal classification requires confirmation by NMR-based elucidation	Tumor cell lines	Cytotoxic and genotoxic activity; apoptosis induction	[19]
Narciclasine	Amaryllidaceae alkaloid (isocarbostyril class)	Experimental tumor cell models	Inhibition of tumor cell proliferation via eukaryotic peptide elongation factor inhibition and protein synthesis	[35]
Hemanthamine	Hemanthamine-type alkaloid	Tumor cell models reported in the family literature	Inhibition of protein synthesis at the ribosomal level; cytotoxic activity	[15]
Montanine-type	Montanine-type alkaloids	In vitro tumor models	Antiproliferative effect observed in cell viability assays; discrete molecular target not yet established in the primary literature	[20]

## Data Availability

No new data were created or analyzed during this study. Data sharing is not applicable to this study.

## Supplementary Materials

The following supporting information can be downloaded at: https://www.mdpi.com/article/10.3390/ijms27198466/s1.

## Abbreviations

The following abbreviations are used in this manuscript:

AKT	Protein kinase B
DNA	Deoxyribonucleic acid
GC–MS	Gas chromatography–mass spectrometry
LC–MS	Liquid chromatography–mass spectrometry
MAPK	Mitogen-activated protein kinase
NF-κB	Nuclear factor kappa B
NMR	Nuclear magnetic resonance
PI3K	Phosphoinositide 3-kinase
ROS	Reactive oxygen species
