# Supplementary material for: Alkaloids Reported in the Genus Griffinia (Amaryllidaceae): Phytochemical Evidence and Current Limitations in Anticancer Interpretation
Source: Int J Mol Sci. 2026 Sep 23;27(19):8466. doi: 10.3390/ijms27198466 (PMC13665542; doi:10.3390/ijms27198466)
Supplement: Supplementary file 1 [file ijms-27-08466-s001.zip › ijms-4393006-supplementary.pdf]

Supplementary Table S1. Molecular targets and signaling pathways associated with the anticancer effects of Amaryllidaceae alkaloids relevant to the structural classes reported in *Griffinia*.

Alkaloid	Experimental model	Molecular target/protein	Signaling pathway/process	Biological outcome	Evidence in *Griffinia*?	Reference
**Lycorine**	HepG2	Caspase-3, caspase-9	Mitochondrial apoptosis	Apoptosis	**No – contextual**	[9]
**Lycorine**	Osteosarcoma	β-catenin; ERK1/2; AKT-related signaling	Wnt/β-catenin; ERK/MAPK; PI3K/AKT	Reduced proliferation/tumor growth	**No – contextual**	[36]
**Lycorine hydrochloride**	Gastric cancer	FBXW7, MCL1	FBXW7–MCL1 axis	Reduced proliferation/apoptosis	**No – contextual**	[37]
**Lycorine**	Colorectal cancer	IDH1	IDH1 acetylation/mitochondrial dynamics	Mitochondrial dysfunction	**No – contextual**	[33]
***G. gardneriana* alkaloids**	Experimental models	Caspase-3-associated response	Apoptotic signaling	Cytotoxicity/genotoxicity/apoptosis	**Yes – direct**	[8]
